# Assessing the impact of urban planning policies on renewable energy: A case a China using the DID estimation model

**DOI:** 10.1016/j.heliyon.2024.e27099

**Published:** 2024-02-24

**Authors:** Rui Ge, Shan Xu, Mirzat Ullah, Peter Mark

**Affiliations:** a Office of International Cooperation and Exchange, Huaiyin Institute of Technology, Huaian, Jiangsu, 223003, China; b Shandong Energy Power Sales Co., Ltd., Jinan, 250014, China; c Graduate School of Economics and Management, Ural Federal University, Yekaterinburg, 620002, Russia; d College of Finance and Economics, Anhui University, China

**Keywords:** Urbanization, Smart city pilot program, Innovation, Financial and ecological development

## Abstract

The ongoing pace of urbanization poses a substantial obstacle to the concurrent progress of both financial and ecological development. Recognizing this challenge, governments globally are formulating cutting-edge strategies for urban renewal to ensure the long-term sustainability of cities. In this context, we employ a difference-in-differences model to scrutinize the intricate relationship between smart cities and the growth of renewable energy, utilizing the Chinese smart city pilot program as a pertinent experiment. This analytical approach provides novel insights into the underlying reasons behind this correlation. The research yields three noteworthy findings. Firstly, it underscores the indispensable role of pilot initiatives in smart cities for advancing the cause of renewable energy. Secondly, the study reveals a positive and beneficial interplay between creativity, economic inclusion, and the utilization of technological innovation in experimental urban programs, suggesting a potential multiplier effect. Thirdly, the local context significantly influences the impact of smart city pilots, with the dissemination of renewable energy being particularly effective in resource-rich, metropolitan, and coastal cities. Observable impacts of current smart city experiment on energy security and sustainable development are already apparent. The research findings contribute fresh perspectives to the complex challenges of sustainable energy production and urban planning, especially in developing countries like China.

## 1 Introduction

Since the initiation of the improvement and opening-up program, China's urbanization has undergone a substantial increase, witnessing a growth in the urban population from 62% in 1980 to 240% in 2022. This surge has played a pivotal role in fostering financial development and elevating the living standards of urban residents. Nevertheless, it has also given rise to a host of significant challenges commonly referred to as "urban diseases." These challenges encompass escalated traffic congestion, a decline in the quality of public services, environmental degradation, and a scarcity of affordable housing, as elucidated by Ref. [1]. The repercussions of these issues are far-reaching, impacting environmental conditions, citizens' health, and overall quality of life. Numerous studies have independently arrived at a shared conclusion: the exacerbation of environmental issues can be chiefly attributed to the rapid urbanization in China, dominated by heavy industries and characterized by high inputs and emissions, as asserted by Ref. [2]. In the context of a developing economy like the People's Republic of China, the annual escalation in energy consumption is substantial, as highlighted by Ref. [3]. Regrettably, this prevalent form of city growth has significantly impeded the trajectory of sustainable development in China.

Confronted with these increasingly challenging circumstances, there is an urgent need to enhance China's core city systems through the implementation of more proficient institutions and technology. Simultaneously, the conventional urban administration system must be further optimized, with a particular focus on supporting clean energy consumption, as underscored by Ref. [4]. Additionally, there is a pressing need to refine China's traditional urban management strategy. Taking into consideration the myriad challenges faced by major cities in China, this study proposes an urban planning policy centered around the utilization of renewable energy. To analyze the efficacy of this proposed policy, a novel econometric estimation model, namely the difference-in-difference (DID) approach, is employed. The objective is to provide a comprehensive framework for addressing the complex interplay of urbanization, environmental concerns, and sustainable energy practices in China's evolving urban landscape.

The world is taking notice of China's tremendous accomplishments in urbanization as the nation enters a new stage of its growth. Fast urbanization has led to undesirable side effects, or "urban diseases," including the depletion of natural resources and the deterioration of the built environment while significantly contributing to economic development[5]. When these challenges receive attention, cities' chances of sustaining sustainable growth could be much higher. The Chinese government has made "vigorously fostering a green economy and strengthening industries such as clean energy, ecology, and green infrastructure enhancement" a central development goal in the country's 14th Five-Year Plan to reconcile financial development with resource preservation and ecological[6]. This goal was established to balance economic growth with environmental responsibility and resource conservation. The report presented at the 20th National Congress of the Communist Party of China identified opportunities for improvement in total energy consumption and the intensity of that use. The report also emphasized the need to lower dependency on fossil fuels and encourage energy usage that is carbon-aware, environmentally responsible, and resourceful[7].

In 2012, China made its first real effort toward these goals with the introduction of the Smart City Pilot Program. New approaches to solving urbanization problems are emerging with the rise of "smart cities," which seek to promote social growth by enhancing technological advancement and boosting collaboration between regions. The creation of "smart cities," which emphasized resource preservation and environmental protection, is suggested as a solution to the issues brought on by fast economic expansion[8,9]. Long-term, this will result in environmentally responsive and carbon-efficient urban growth. Since the turn of the century, increased urbanization has led to several issues, including smog, sewage, and sulphide emissions. Worries about environmental degradation negatively impact people's health and quality of life. Many countries, including China, have implemented smart city pilot projects to solve these problems and promote ecologically responsible urbanization. They aim to maximize their resources while achieving sustainable economic and environmental progress[10].

Pilot programs for "smart cities" use cutting-edge technology and the growth of digital infrastructure to promote environmentally friendly urban growth. The internet, the blockchain technology, and the rise of the Internet of Things are just a few examples of these developments and innovations. New technology deployment and the improvement of digital infrastructures are both energy-intensive processes. traditional fuel-driven power generation is incompatible with the goals of smart cities due to the complexity of pollutant emissions and the costly nature of energy consumption brought on by traditional fossil fuels[11]. Using renewable energy would help prevent environmental harm from increasing along with economic growth. Cheap levels of byproduct emissions and cheap cost are related to the production of clean energy, both of which support the growth of urban areas that are environmentally conscious. Therefore, smart city pilots will fuel the growth of renewable energy. Research of 20 developing countries between 1991 and 2012 found that both FDI and stock markets helped use renewable energy[12,13]. It discovered that money generated from implementing carbon trading regimes may be utilized to increase access to renewable energy. Because most research has concentrated on economic concerns, including few studies have examined the effects of urban planning regulations on developing clean energy. Our investigation into the relationship between smart city pilot programs and the growth of renewable energy sources will provide fresh insights for urban planning in the future.

In accordance with this objective, the present study makes several substantive contributions to the existing literature on various fronts. Firstly, it furnishes empirical evidence establishing that smart city pilot projects accelerate the adoption of renewable energy sources. These findings align with the seminal work conducted by Ref. [14]. The development of innovative cities can be robustly anchored in digital technology. The implications of the study suggest that the utilization of digital technologies facilitates the creation of smart cities. Overcoming associated challenges, such as the requirement for investment capital, technical support, and essential infrastructure development, is crucial. Access to advanced digital technologies enables smart cities to fully exploit their strengths, fostering industrial convergence, smart manufacturing, and sustainable innovation, as articulated by Ref. [15]. The increasing reliance of smart cities on digital technology enhances resource mobilization and utilization, thereby fostering an environment of openness and transparency. However, addressing long-term investment projects in clean energy and innovation, which inherently involve uncertainties, poses challenges in providing requisite financial support. Smart cities can surmount these challenges by leveraging their strengths to drive sustainable energy innovation and construction efforts, as suggested by (H. [16]). Moreover, modern technology contributes significantly to the growth of renewable energy, highlighting the imperative of equitable financing and the utilization of cutting-edge technology for the development of environmentally friendly energy in smart cities.

Furthermore, this research contributes to bridging the gap in the literature by elucidating that smart cities and the growth of renewable energy sources exhibit a multiplier effect, with the presence of digital technology acting as a moderator. This effect is particularly pronounced in the context of China's burgeoning economic condition. Incorporating the financial sector and new technologies becomes imperative, as emphasized by (J. [17]). The study employs data from 285 Chinese cities spanning the period from 2009 to 2022, deploying a difference-in-differences model to scrutinize the impact of smart city pilot programs on the proliferation of renewable energy and the underlying mechanisms at play. The findings of this investigation have broad applicability. The use of the difference-in-differences model serves as a foundational step in examining the connections between innovative city pilot projects and the growth of renewable energy sources, as highlighted by Ref. [18]. Given China's status as the world's foremost carbon dioxide producer and the exacerbation of its environmental issues, the study positions China as a case study offering valuable insights for urban policy formulation and renewable energy growth in other developing nations. According to our study, digitalization possesses the potential to enhance the benefits of smart cities, with inclusive financial systems and cutting-edge technology serving as valuable mediators, echoing the sentiments of [19]. Municipal officials and lawmakers now have a repertoire of options for developing innovative city pilot programs conducive to sustainable urban growth. The study concludes by examining urban heterogeneity in light of China's vast geography and disparate economic growth, asserting that valuable lessons can be gleaned from China's diverse urban features. In seeking to enhance pilot plans for smart cities and promote the growth of renewable energy, our study aspires to introduce fresh theoretical perspectives, as articulated by Ref. [20].

The remainder of this essay is organized as follows. The study assumptions and the policy background are thoroughly explained in Section 2. We go more into these strategies in Section 3. The next part will go into further depth about the empirical results and robustness verification. The examination of heterogeneity is in Section 5. A summary of the conclusions and their political ramifications is provided in Section 6.

## 2 Literature review

In 2008, IBM introduced the concept of "smart cities" with the aim of promoting environmentally responsible growth in large urban areas. This innovative system integrated diverse operating networks, infrastructures, and cityscapes into a cohesive framework. Our argument posits that the incentive for competent city pilots to advocate for clean energy development stems from resource-based theory. This theory, as elucidated by Ref. [21]. in 2022, posits that companies possess a variety of natural and intangible resources that enable them to develop distinctive capabilities. Although formulated in the 1970s, this theory has been applied at the municipal level, where cities leverage a diverse range of resources to foster long-term sustainability. However, the commitment to environmentally friendly energy is hindered by high upfront costs and volatility, as noted by Ref. [22]. in 2021. China's smart city initiatives strategically address various facets to enhance urban living, particularly emphasizing energy conservation and traffic management. Implementation efforts involve the deployment of intelligent transportation systems (ITS) to optimize traffic flow, minimize congestion, and integrate real-time data from sensors and cameras for improved signal timing and route planning. Smart parking solutions are also incorporated to address parking-related traffic issues. Public transportation is enhanced through technologies such as real-time tracking, smart scheduling, and contactless payment systems, alongside the introduction of eco-friendly modes like electric buses and shared mobility solutions (Lin et al., 2022).

### 2.1 Developing test projects for smart cities and alternative forms of energy

Given the complexity of planning and executing these initiatives with renewable energy, external resources and astute resource management become crucial. Complementary technology expands the range of resources available to smart cities, potentially facilitating the development of renewable energy facilities, as articulated by (W. [23]). Present-day "smart cities" leverage cutting-edge technology and data analytics to enhance the integration and coordination of various urban components, thereby achieving higher energy efficiency and productivity (EEP). The model's primary objectives are to balance environmental and urban energy needs, enhance urban operations' effectiveness, promote sustainable growth, and improve overall living standards, as emphasized by Ref. [24].

The pursuit of increased efficiency in city operations was a primary goal when IBM introduced the concept of a "smart city" in 2008. Since then, it has garnered widespread interest globally, inspiring governments to initiate their own smart city development initiatives, as highlighted by Ref. [25]. Recognizing the significance of smart cities, the Chinese government has prioritized their development. The Smart City Pilot Project (SCPP) was initiated in 2012, comprising 90 communities established by the Ministry of Housing and Urban-Rural Development (MOHURD) to oversee and promote long-term smart city growth. Subsequent waves of smart city pilots were announced in 2013 and 2014, emphasizing the need for smart city planning frameworks aligned with the National Indicator System of Pilot Smart City. The SCPP mandates the development and implementation of facilities and technical tools for industrial sewage discharge, air quality monitoring, energy smart management, urban construction management, and specific applications. This system aims to enhance the city's energy efficiency assessment, monitoring, administration, and management by establishing more effective standards, as articulated by (L. [26]). Key initiatives include the implementation of smart grids for optimized energy distribution, monitoring and control of energy consumption, and integration of renewable energy sources like solar panels and wind turbines into urban infrastructure.

Data plays a pivotal role in governance, with analytics and artificial intelligence optimizing city operations and resource allocation. Smart city platforms facilitate the integration and analysis of data from various sources, including traffic sensors, energy meters, and environmental monitoring devices [27]. The integration of information processing theory is proposed to elaborate on how smart city development influences the utilization of renewable energy. Certain digital technologies in smart cities are posited to enhance information processing capabilities and reduce the cost of data transfer across cities, potentially attracting external interest and investor confidence in renewable energy projects. This, in turn, is argued to set smart cities apart, as their enhanced information processing skills contribute to the success of green initiatives by reducing information disparity and increasing resource efficiency. Incorporating information processing theory and resource-based theory into smart city planning is suggested to be beneficial for the successful completion of renewable energy projects. In light of these insights, the study proposes the hypothesis:

H1 A pilot program for smart cities provides assistance for the development of sustainable energy sources.

### 2.2 The mediating and reconciliatory function that innovation plays

Achieving progress in the development of sustainable energy is contingent upon leveraging cutting-edge technology. The conceptualization of the smart city pilot strategy as an urban preparation policy characterized by ecological constraints was introduced by (X. [28]). According to Porter's framework, smart cities have the potential to pool resources to yield practical innovations. Empirical evidence derived from panel data spanning thirty provinces in China from 2003 to 2017 revealed a robust "Porter's effect," indicating that environmental constraints significantly influence innovation. A survey conducted by (H. [29])among urban residents in China affirmed that government regulations play a pivotal role in fostering green innovation. Technological advancements, as proposed by Ref. [30], could lead to the discovery of new ecological energy sources and the removal of existing barriers to energy production. Similarly, a study analyzing panel data from 33 countries spanning the period 1990 to 2015 concluded that environmental regulations significantly promote the adoption of renewable energy, chiefly driven by advancements in green innovation.

Considering the impact of environmentally friendly technology on electricity consumption reduction and increased reliance on renewable sources, we propose the hypothesis that smart systems, incorporating sensors for waste level monitoring and optimizing collection routes, can enhance waste management efficiency, thereby reducing fuel consumption. Intelligent street lighting, utilizing systems that adjust brightness based on real-time conditions, not only contributes to energy savings but also incorporates adaptive lighting and advanced safety features. Prioritizing environmental monitoring, the integration of sensors for real-time tracking of air and water quality, as highlighted by Ref. [31], facilitates swift responses to environmental concerns and enables the development of early warning systems for natural disasters. Despite these positive developments, challenges persist. Privacy and security concerns associated with data collection and usage, infrastructure costs, and the necessity for substantial investments demand careful consideration. Addressing potential cybersecurity threats linked to interconnected smart systems and ensuring inclusivity are ongoing priorities. For the latest and most accurate information, it is recommended to consult official government sources and stay informed through recent news updates.

H2a The introduction of innovative technology acts as a catalyzing agent to speed up the development of renewable energy sources in smart cities.

The crux of innovative city pilot projects lies in the utilization of digital technology to seamlessly connect various systems while demonstrating a high level of sustainability. This approach aims to overcome obstacles hindering the construction of smart cities by enabling the connectivity of diverse subsystems to enhance overall capacity, as discussed by Ref. [32]. However, it is crucial to acknowledge that the use of such digital technology entails the consumption of significant amounts of power. To address this energy consumption concern, leveraging renewable resources becomes a viable solution to generate environmentally friendly electricity at a cost-effective rate. Establishing a platform that facilitates open and transparent information disclosure, coupled with an energy management system enabled by digital technology, plays a pivotal role in identifying external social and public pressures on the environment. This, in turn, enables the adoption of preventive measures. Furthermore, digital technology can be effectively employed to optimize the utilization of scarce resources and ensure their fair distribution, as highlighted by Ref. [33]. Building upon the insights derived from this study, we propose hypothesis:

H2b Digital technology serves as the driving force behind the expansion of sustainable energy.

Experiments within smart cities, leveraging digital technology, are underway to explore and implement innovative solutions in this realm.

### 2.3 The function that inclusive finance plays as a mediator

The finance sector plays a pivotal role in driving the expansion of renewable energy sources by identifying and fostering the development of environmentally conscious innovations. Inclusive finance, which facilitates the seamless movement of capital within and across businesses, is integral to the overall financial system of our country. Given the substantial financial assistance required for the progress of sustainable energy development, the imperative for fair financing becomes paramount. The conclusion drawn is that fair funding is indispensable for the sustained growth of renewable energy sources and the promotion of environmentally responsible practices. As highlighted by Ref. [34], improved access to financial resources is a key factor enabling the continued growth of the green economy. Building upon these findings, we propose hypothesis:

H2c The utilization of inclusive finance in innovative city experiments serves as a facilitator for beneficial mediation.

This suggests that inclusive financial mechanisms can play a crucial role in supporting and mediating the implementation of sustainable and environmentally friendly initiatives within smart city experiments.

## 3 Method, materials and model

This study sourced historical data from the official data repository, namely the "Chinese Urban Statistics Yearbook," which served as the principal reservoir of city-level statistics spanning the period from 2009 to 2022. This dataset was chosen to encompass the influence of both global financial crises and health-related crises on the variables under investigation. Moreover, a meticulous manual review of each annual report was undertaken to address data gaps, culminating in the collection of primary data from a total of 4135 participants for this research endeavor. Subsequent to a thorough examination, we refined and finalized our dataset, ultimately incorporating 3897 observations for inclusion in this study. This refinement process involved the implementation of a revised coding technique to ensure data accuracy and reliability.

### 3.1 Variable selection

The dependent variable in our study, denoted as CE (Clean Energy), is characterized by the quantity of clean energy installations within each province and the aggregate electricity consumption across urban and rural areas. The data utilized for this analysis was extracted from the China Electricity Statistics Yearbook. Conversely, a Boolean independent variable, denoted as SCPP (Smart City Pilot Policy), was incorporated into our study. The value of SCPP is binary, being assigned a value of one if the location under consideration is officially recognized as a smart city and zero otherwise. It is noteworthy that the Chinese Ministry of Housing and Urban-Rural Development maintains a roster of pilot smart cities, categorizing a location as a "Smart city" if listed. The selection of ninety sites in 2012 for testing purposes was subsequently expanded in 2018 and more recently in 2022.

Furthermore, our research incorporates several control variables to ensure a comprehensive analysis. These variables include gross domestic product (GDP), industrial structure (measuring the sectorial contribution in terms of secondary and tertiary sectors), fixed capital investment (FCI), road space per capita (RS), and green space per capita (GS). The selection of these control variables aligns with the literature, specifically referencing the work of Yang et al. [35]. To mitigate the potential impact of unobserved components on the regression results, our estimation technique involves controlling for province and time effects within the urban clustering. This strategic approach seeks to eliminate the influence of unidentified factors on the outcomes of the regression analysis. The control variables adopted for this purpose include GDP per capita, FCI, industrial structure (IS), green space per capita (GS), and road space per capita (RS). The incorporation of province and time effects in the urban clustering serves to enhance the robustness and reliability of our regression results by accounting for unobservable factors that may affect the study variables.

### 3.2 Model selection

We employed a difference-in-differences model to examine the correlation between smart cities and the growth of renewable energy, utilizing the Chinese smart city pilot program as a suitable experimental context. This novel approach contributes valuable insights into understanding the underlying reasons for this correlation. In the third phase of our analysis, we assessed the impact of certification on hospital efficiency. Certification, in this context, is viewed as an event that can significantly alter the trajectory of an organization, potentially influencing its overall performance. The difference-in-difference methodology was applied to discern changes in the efficiency of certified hospitals in comparison to non-certified ones, while accounting for time-varying variables. A fundamental assumption inherent in this approach is that all other temporal factors affecting hospital efficiency had an equivalent impact on both the intervention group and the control group. We posit that any uncontrolled changes over time affected all hospitals uniformly. To address the issue of serial correlation in our difference-in-difference panel analysis, we implemented individual regressions for each of the six comparison periods, as outlined by Ref. [36]. This approach allowed us to explore the impact of certification not only within a fixed post-period but also as an evolving effect over time.

Consistent with the methodology proposed by Ref. [37], we utilized a truncated regression model, recognizing that efficiency is bounded at 1, implying it cannot exceed this value in equation (1). The model we present here in the form of an equation, estimating the difference-in-difference model, was employed to investigate the influence of smart cities on hospital efficiency.

(1) $${CE}_{i,t}=\alpha_{0}+\beta_{0}{SC}_{i,t}+\sum_{i=1}^{5}\gamma_{i}control+\varepsilon_{i,t}$$

The equation for clean energy incorporates the variable SCPP, representing solar concentration. In establishing our control variables, we drew upon previous research in the field. These controls include per capita gross domestic product, energy and environmental performance, and the industrial structure ratio (ISR: tertiary to secondary sector ratio). Examples of such metrics encompass measurements like the amount of parkland and road space allocated per person. To mitigate the impact of unobservable factors on the regression outcomes, we incorporated provincial and temporal effects during the urban clustering process. Given that the conventional Difference-in-Differences (DID) model, designed for single-batch policy analysis, is ill-suited for China's SCPP's three-batch configuration, we employed a multi-period DID model. This approach optimally addresses the challenge, allowing us to discern the effects of policy adjustments across various batches on the entire system. The utilization of a multi-period DID model in this study enhances the credibility of the policy assessment. It enables a more accurate evaluation of the impact that SCPP has on Energy and Environmental Performance (EEP) in urban areas. This methodological choice ensures a comprehensive and nuanced understanding of the influence of varying batches of policy adjustments on the overall outcome in equation (2).

(2) $$EEPI_{it}=\alpha_{0}+\alpha_{1}did_{it}+\lambda X_{it}+v_{i}+\mu_{t}+\varepsilon_{it}$$

The significance of the dependent variable in this context, denoted as [variable], cannot be overstated for my research objectives. In this instance, the SCPP variable is replaced by the explanatory variable. The crux lies in assessing the magnitude of coefficient 1; if it significantly exceeds zero, it indicates that the pilot plan has substantially enhanced Energy and Environmental Performance (EEP) in the respective city. The term "here" encapsulates the perpetual influence of the city, the ongoing impact of the passing year, and the inherent unpredictability associated with the disturbance connotation. Recognizing the intricate nature of factors influencing urban EEP, the author proposes a moderator. At Level 1 of GDP (economic development), there is potential for significant impacts on EEP to be discerned. Robust economic growth often correlates with advanced technology levels and structures within the industrial sector, promoting energy efficiency and reducing pollution. This correlation is typically gauged against gross domestic product per capita. While rapid economic growth may exacerbate pollution issues initially, it tends to enhance environmental standards in later stages in equation (3). The model is refined by introducing the squared component of real GDP per capita, capturing the non-linear nature of this relationship.

(3) $$M_{it}=\beta_{0}+\beta_{1}did_{it}+\lambda X_{it}+v_{i}+\mu_{t}+\varepsilon_{it}$$

While some scholars advocate for a three-step mediation effect model, commonly employed in psychological research, applying it directly to an economic study may overlook endogeneity issues. Thus, the mechanism in this study aligns with the research strategy, validating impacts of explanatory factors on fundamental variables within the mechanism. A transmission channel exists if coefficients 1 and 1 are significant—this being the sole condition for such a channel. Fully developed models encapsulate the mechanisms as follows in equation (4):

(4) $$EEPI_{it}=\varphi_{0}+\varphi_{1}M_{it}+\lambda X_{it}+v_{i}+\mu_{t}+\varepsilon_{it}$$

In the provided graphic, a depiction of the mechanism variable, alternatively known as the economic concentration variable and the drive to innovate, is presented. The variable denoted as "egg" illustrates the value of all secondary and tertiary industrial production expressed as a ratio to the size of the urban administrative area. The units for "egg" are 10,000 yuan per km^2^, representing the economic concentration. Additionally, the graphic includes the Inno index, which assigns a ranking to each city based on its level of inventiveness. This index serves as a measure of the drive to innovate within each city.

## 4 Preliminary analysis and results

### 4.1 summary statistics

Table 1 presents the descriptive statistics pertaining to the data under consideration. Specifically, the metrics for total factor productivity are delineated, with the mean, minimum, and maximum values being recorded as 15.584, 9.377, and 18.081, respectively. These figures suggest a notable degree of overall productivity within the factor production framework.

**Table 1:** Presents descriptive data as well as a test of correlation.

Variable	Treated			Control		
	Obs	Mean	Std. Dev.	Obs	Mean	Std. Dev.
CE	2661	21.153	2.696	3897	20.850	2.597
SCPP	2672	1.651	1.477	3897	10.07	2.117
GDP	2672	20.772	1.645	3897	20.426	1.598
EEP	2672	26.428	1.959	3897	25.798	1.813
ISR	2672	1.968	1.510	3897	1.962	1.602
GS	2665	34.311	50.626	3897	22.717	29.578
RS	2658	7.374	23.183	3897	4.329	4.182

Note: CE, GDP, EEP are treated as natural logarithms. ISR, GS, RS are proportions.

**Fig. 1:**
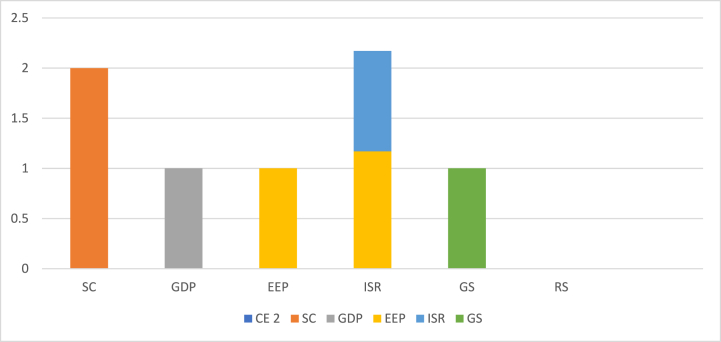
Shows the correlation of different parameters.

Furthermore, an observation that can be drawn from these statistics is the potential imbalance in the application of circular economy techniques across different cities. This indicates that the adoption and implementation of circular economy practices may vary significantly among the studied locations in Fig. 1. Additionally, disparities between enterprises, as evidenced by other relevant factors, are also discernible, highlighting the presence of inequities within the operational landscape.

#### 4.1.1 Correlation matrix

**Fig. 2:**
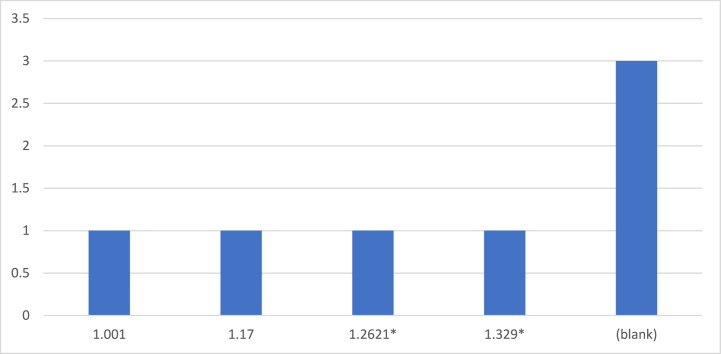
Shows the CEP significantly reduce a company's total factor productivity.

Table 2 showcases the outcomes of the correlation tests conducted in our study. Our analysis reveals a significant association between Corporate Environmental Performance (CEP) and a company's total factor productivity, thereby substantiating our initial hypothesis, denoted as H1. This finding suggests that the implementation of CEP may indeed have a noteworthy impact on reducing a company's overall productivity in Fig. 2. Furthermore, the results of the multicollinearity test are conveyed through the variance inflation factor, yielding a value of 0.59 (0.59). This information signifies that our model exhibits a low level of multicollinearity. The data provided supports the conclusion that there is no substantial correlation among the predictor variables in the model, enhancing the reliability of our analytical framework.

**Table 2:** Describes the correlation test.

	CE	SC	GDP	EEP	ISR	GS	RS
**CE**	1.000						
**SC**	0.249*	1.000					
**GDP**	0.480*	0.426*	1.000				
**EEP**	0.339*	0.432*	0.606*	1.00			
**ISR**	0.200*	−0.203*	0.009*	0.070	1.000		
**GS**	0.336*	0.300*	0.696*	0.2620*	0.887*	1.000	
**RS**	0.344*	0.338*	0.735*	0.329*	0.794*	0.878*	1.000

### 4.2 Hypothesis testing for parallel trend (PT)

The Differential-in-Differences (DID) approach is fundamentally rooted in the assumption of a parallel trend, constituting its foundational premise. This posits that the sole factor influencing the dependent variable in response to a policy change is the impact of the policy itself. The results of the parallel trend test unveil that the coefficients associated with the policy remained closely proximate to zero in the years preceding the implementation of the Smart City (SC) initiative. This observation underscores the imperative nature of satisfying the parallel trend assumption to prevent skewed evaluations of policy impacts, as highlighted by Dargan et al. (2020). The discerned proximity to zero in the pre-implementation years signifies the alignment of the parallel trend hypothesis, indicating a high degree of congruence between the treatment group (subject to the policy) and the control group (not subject to the policy) during this period. This congruence substantiates the robustness of the parallel trend assumption and reinforces the reliability of employing the DID methodology to assess the genuine effects of the Smart City policy.

### 4.3 Investigation of the baseline estimation

Table 3 presents the results derived from the regression analysis conducted on the control years and control components. The regression outcomes distinctly indicate that the implementation of Smart City (SC) initiatives may substantially enhance the proliferation of renewable energy within urban areas, thereby corroborating the validity of hypothesis H1. For instance, when considering equation (3) as an illustration, the coefficient associated with the variable CS is determined to be 0.081, achieving statistical significance at the 10% level (Lacal Arantegui & Jäger-Waldau, 2018). This empirical evidence underscores that smart city pilot programs play a constructive role in advancing the research and development of renewable and clean energy within urban environments.

**Table 3:** Presents the baseline findings of the regression.

Variables	(1)	(2)	(3)
	CE	CE	CE
SC	2.109***	1.569***	1.078*
	(1.051)	(1.049)	(1.045)
GDP			1.825***
			(1.045)
EEP			1.319***
			(1.029)
ISR			−1.168***
			(1.045)
GS			1.004***
			(1.002)
RS			1.009***
			(1.003)
C	30.628***	20.034***	−4.068***
	(1.029)	(1.064)	(2.468s)
Province	Yes	Yes	Yes
Year	No	Yes	Yes
Observations	4122	4122	4098
R-squared	1.424	2.591	1.714

Note that the symbols ***, **, and * indicate significance at 1%, 5%, and 10%, respectively.

### 4.4 Examination of robustness

#### 4.4.1 The trial with the placebo

A placebo test was meticulously conducted, and the resulting estimated coefficients and corresponding p-values were documented based on a random sample of 500 individuals. These deliberate measures were implemented to ensure the reproducibility of the results and to mitigate the potential influence of unobserved factors on the conclusions drawn from the regression analysis. The outcomes of the placebo test revealed that each estimate was consistently lower than one, with their distribution center situated in close proximity to zero. This particular finding raises the possibility that the conclusions derived from our analysis may not be attributed to other factors that warrant thorough investigation. However, it is imperative to acknowledge that such assertions remain speculative and are presented as a theoretical consideration at this stage of the inquiry. Further investigation and scrutiny are essential to gain a comprehensive understanding of any potential confounding factors that might impact the validity of the conclusions drawn from the regression analysis.

#### 4.4.2 Alternative methods of research

**Fig. 3:**
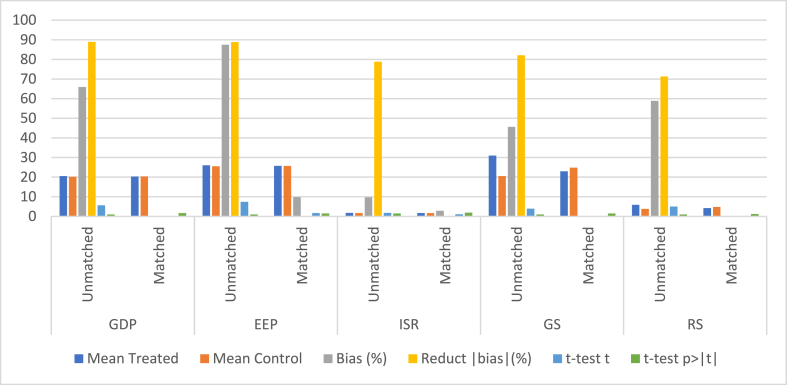
Shows the Using the PSM to check the balance of numbers.

Potential selection bias may exist in Smart City Pilot Policy (SCPP) implementations, wherein factors such as geography, economic conditions, and other city-specific characteristics could influence the authorization of a "smart city" project. To mitigate this bias, we employed the propensity score matching (PSM) technique, as advocated by Ref. [38], to minimize the impact of partiality on the selection process. The PSM approach facilitates the randomization of policies by creating pairs of treatment and control cities with comparable features, thereby reducing the likelihood of bias influencing the selection of "smart city" projects. Pre-legislation, we applied a matching method, specifically the nearest-neighbor technique, to categorize the population into two groups based on urban characteristics in Fig. 3. This process resulted in the refinement of the initial pool of 285 cities to a final sample of 187. The consequential decrease in the standard deviation, as evident in Table 4, signifies a substantial improvement in equivalence between the treatment and control groups. By ensuring comparability through PSM, we successfully mitigated the effects of bias in the selection process, bolstering the robustness and reliability of our analytical framework.

**Table 4:** Check to see the numbers are balanced.

Variable	sample	Mean		Bias (%)	Reduct |bias|(%)	*t*-test	
		Treated	Control			t	p>|t|
GDP	Unmatched	20.544	20.238	65.9	88.9	5.68	1.001
	Matched	20.344	20.379	−7.3		−1.47	1.641
EEP	Unmatched	26.053	25.475	87.5	88.8	7.45	1.001
	Matched	25.761	25.696	9.8		1.71	1.478
ISR	Unmatched	1.748	1.715	9.7	78.8	1.74	1.462
	Matched	1.709	1.702	2.9		1.13	1.898
GS	Unmatched	30.978	20.576	45.6	82.1	3.92	1.005
	Matched	22.992	24.863	−7.4		−1.69	1.488
RS	Unmatched	5.903	3.843	58.9	71.3	5.04	1.001
	Matched	4.227	4.819	−24.1		−1.24	1.219

During the pre-treatment phase, every component must be scrutinized during the pre-treatment stage.

The results of the regression carried out employing the PSM-DID methodology are shown in Table 5. The findings agree with the information shown in Table 3, which suggests that the return results have not changed.

**Table 5:** Presents the findings obtained using PSM-DID in the baseline regression.

Variables	(1)	(2)	(3)
	CE	CE	CE
SC	1.725***	1.022	1.112**
	(1.065)	(1.058)	(1.052)
Constant	20.645***	8.896***	2.852***
	(1.035)	(1.073)	(1.648)
Control	No	No	Yes
Province	Yes	Yes	Yes
Year	No	Yes	Yes
Obs	3053	3053	3038
R-squared	1.378	1.623	1.718

Note that the symbols ***, **, and * indicate significance at 1%, 5%, and 10%, respectively.

### 4.5 Examination of the mechanics

In order to assess the efficacy of sustainable development policies as moderators and to investigate the role of digitalization as a mediator, we constructed an index gauging green innovation and inclusive financing. Adhering to the criteria established by the Chinese Patent Law [39], environmentally responsive findings were classified as either innovations or utilities. The logarithm GUP represents the count of green utility innovation applications, while the logarithm GIP corresponds to the count of green invention innovation applications, both denoted as GIP. This component is referred to as SA, with the ratio of broadband connections per 10,000 households serving as a proxy for the prevalence of digital technology, as outlined by Ref. [40]. These specific numerical values were directly extracted from a database maintained by the China Research Data Service. Additionally, the inclusive finance statistics (IF) were derived from research on financial inclusion conducted at Peking University in China [41].

Table 6 presents the results of experiments conducted to ascertain both moderating and mediating effects. Both green innovative utility technology (1.193, p 0.001) and green innovative technology (1.234, p 0.001) exhibit positive influences on the mediation process, aligning with our H2a hypothesis. Furthermore, the inclusive finance index demonstrates a significant positive mediating effect (H2c: 3.731, p 0.001), and the digitization of society enhances the beneficial impacts of smart cities (H2b: 1.023, p 0.001). In conclusion, our findings suggest that green innovation, digitalization, and equitable funding serve as crucial pathways for smart cities to explore in order to augment their utilization of renewable energy.

**Table 6:** Examination of the mechanics.

Variables	(1)	(2)	(3)	(4)
	GIP	GUP	CE	IF
SC	1.233***	1.191***	1.233*	3.731***
	(1.038)	(1.033)	(1.055)	(1.438)
SC × BA			1.022***	
			(1.004)	
BA			1.004***	
			(1.005)	
Constant	−31.113***	−27.010***	−1.822***	−298.038***
	(1.404)	(1.351)	(1.571)	(6.283)
Control	Yes	Yes	Yes	Yes
Province	Yes	Yes	Yes	Yes
Year	Yes	Yes	Yes	Yes
Observations	4079	4079	3482	3486
R-squared	2.823	2.848	2.736	2.986

Note that the symbols ***, **, and * indicate significance at 1%, 5%, and 10%, respectively.

### 4.6 A study of the diverse characteristics of cities

It is imperative to consider the potential impact of the number of cities on research and development efforts related to renewable energy. In this context, the sample cities under scrutiny have been systematically categorized based on distinct criteria, as elucidated by (T. [42]). These criteria encompass city size, distinguishing between large cities (LC) and small cities (NC), as well as geographical attributes such as coastal (CC) or inland (NC) locations. Additionally, the classification extends to cities with a resource-based (RC) or non-resource-based (NC) nature. The differentiation between major and minor urban centers is demarcated by the designations "large cities" and "small cities," respectively. This classification is contingent upon various factors, including population size, geographic characteristics, and adherence to the Chinese government's list of resource-based cities. The outcomes of a rigorous regression analysis focusing on urban heterogeneity are meticulously detailed in Table 7.

**Table 7:** A breakdown of the diversity of the cities.

Variables	(1)	(2)	(3)	(4)	(5)	(6)
	CCs	Non-CCs	LCs	Non-LCs	RCs	Non-RCs
	CE	CE	CE	CE	CE	CE
SC	1.499***	−1.004	1.132*	−1.044	1.085*	1s.105
	(1.132)	(1.046)	(1.069)	(1.057)	(1.052)	(1.082)
Constant	−4.388**	−3.401***	−1.830	−3.495***	−4.598***	−1.786
	(2.423)	(1.512)	(1.755)	(1.749)	(1.533)	(2.041)
Control	Yes	Yes	Yes	Yes	Yes	Yes
Province	Yes	Yes	Yes	Yes	Yes	Yes
Year	Yes	Yes	Yes	Yes	Yes	Yes
Observations	3642	557	1547	2552	3354	845
R-squared	1.704	1.725	1.673	1.793	1.711	1.778

Note that the symbols ***, **, and * indicate significance at 1%, 5%, and 10%, respectively.

Our comprehensive investigation leads us to a discerning conclusion: the Smart City (SC) paradigm significantly influences the expansion of the clean energy sector, particularly in coastal (CC), large (LC), and resource-based (RC) cities. This is substantiated by the regression coefficients attributed to the SC variable. Specifically, the SC coefficient for coastal cities stands notably at 1.498, attaining statistical significance at the 1% threshold [43]. Similarly, at the tenth percentile level of statistical significance, the SC coefficient for large cities is recorded at 1.133. Furthermore, at the 10% significance level, the SC coefficient for resource-based cities is computed as 1.086. The imperative for developing sustainable energy sources is underscored, and the nuanced nature of this necessity is underscored by the diverse contexts presented by different cities. Each city's unique characteristics and classification play a pivotal role in shaping the approach and emphasis placed on the development of renewable energy sources.

### 4.7 Discussion

This research endeavors to investigate the positive influence of smart cities on the development of clean energy through the utilization of advanced digital technology. In the preliminary phases of smart city pilot studies, the integration of digital technology has been observed to enhance information processing capabilities, secure resources for clean energy initiatives, and mitigate information gaps. The study underscores that smart cities play a pivotal role in fostering green innovation and financial inclusion, thereby promoting environmentally friendly technologies and reducing the risk of failures in clean energy projects. It is emphasized that smart cities, armed with sophisticated digital technology, exhibit a heightened propensity for advancing clean energy development. The research also takes into account the diverse geographical landscape of China, highlighting that the success of smart cities hinges not only on policy frameworks but also on aligning city resources, especially in coastal, large, and resource-based urban centers.

In order to contribute to existing research lacunae, the investigation commences by scrutinizing how resource-based and information-processing theories predict the substantial impact of smart cities on the growth of renewable energy. A comparative analysis of the outcomes from our innovative city experiment with those presented by Ref. [44] reveals a notable degree of similarity. Smart cities leverage digital technology to enhance information processing capabilities, facilitating the collection of funds requisite for clean energy projects, thereby encouraging sustainable growth [45]. This is achieved through the elimination of communication asymmetry between local administration and stakeholders, as well as the mobilization of resources[46]. Secondly, the research establishes that the relationship between "green innovation," encompassing both creative and utility innovation, and economic participation can be regulated by the presence of "smart cities." Drawing on the Porter hypothesis, which posits that environmental pressures prompt businesses towards environmentally friendly innovation (S. [46]), this finding is reinforced by evidence from Ref. [47]. Smart cities, through their environmental regulations, specify criteria for companies and communities, thereby encouraging the development of environmentally friendly technologies. These innovations have the potential to expedite clean energy development and contribute to environmentally responsible urban design [48].highlight the necessity for low-cost financing solutions to integrate renewable energy sources into smart cities, aiming to broaden access to financial services and stimulate economic growth, fostering social equity. The research posits that the prevalence of digital technology in metropolitan areas is significantly correlated with a greater inclination towards the growth of renewable energy sources, enhancing the adaptability of cities to meet the demands of renewable energy development [49].

Lastly, a study addressing the diversity of China's cities is conducted to elucidate the reasons behind China's vast size and uneven economic growth. The policy framework is identified as a crucial starting point for implementing innovative city projects, providing the necessary support for infrastructure development. The resource-based theory asserts that effective strategies and policies may derive from a variety of resources, leading to long-term success for individuals and organizations [35]. The research demonstrates that smart city policies exert a significant impact on the expansion of sustainable energy in coastal cities, significant communities, and resource-based communities. The well-established commerce, infrastructure, and technology levels in coastal cities have facilitated the development of renewable energy sources such as nuclear, hydro, and ocean energy[50]. The prevalence of powerful economies established market institutions, and abundant resource reserves in large cities and resource-based communities has led to their increased prominence. Leveraging these resources, smart cities are poised to transition to renewable energy more expeditiously.

## 5 Conclusion and policy recommendations

Smart city initiatives in China encompass a comprehensive suite of measures designed to elevate efficiency, sustainability, and the overall quality of life for urban residents. Among these multifaceted measures, the implementation of intelligent transportation systems takes a prominent role, with the specific goal of mitigating traffic congestion and optimizing public transportation networks. Additionally, there is a strong emphasis on energy-efficient infrastructure, incorporating innovations like smart grids and environmentally friendly building designs, all aimed at curbing overall energy consumption. A pivotal aspect of these initiatives lies in the adoption of data-driven governance. Leveraging advanced analytics and artificial intelligence, city officials seek to optimize various operational facets, ensuring a more streamlined and responsive administration. Waste management processes are intricately woven into smart systems, enhancing efficiency, while adaptive technologies are deployed for intelligent street lighting. A robust environmental monitoring framework, encompassing air and water quality assessments, ensures swift and targeted responses to emerging environmental concerns. Despite the impressive strides, it is incumbent upon stakeholders to address challenges associated with privacy, infrastructure costs, and cybersecurity threats. Navigating these challenges demands meticulous consideration to ensure the sustained success of smart city initiatives. For the most up-to-date and accurate information, reliance on official government sources and recent news updates is strongly recommended.

While considerable progress has been achieved, establishing a definitive link between the expansion of renewable energy sources and the evolution of smart cities remains a subject of ongoing research. Despite a burgeoning body of literature on the topic, conclusive evidence is yet to be presented. The ascendancy of renewable energy sources, with their lower carbon footprint, is particularly advantageous for environmentally conscious populations. To dissect the impact of smart city pilots on renewable energy policies in China, the study employed the Difference-in-Differences (DID) and Propensity Score Matching-Difference-in-Differences (PSM-DID) approaches. The analysis focused on prefecture-level cities spanning the years 2009–2019, considering both 2009 and 2022 as potential benchmarks. A noteworthy discovery was that smart city demonstration projects actively stimulate the establishment of renewable energy sources, underscoring the pivotal role played by digital technology in this context. Conversely, the study emphasizes the importance of a balanced approach, advocating for increased access to financial services and the encouragement of environmentally responsible innovation. Research findings suggest that pilot projects possess the potential to accelerate the adoption of renewable energy, particularly in specific urban settings, hinting at their role in promoting sustainable energy growth and eventual carbon neutrality.

In light of these revelations, the study contributes valuable theoretical insights to the effective planning and execution of smart city pilot projects, particularly in burgeoning economies such as China. Recommendations for improving sustainable energy and smart city pilot programs include expanding the geographic scope of pilot initiatives, augmenting governmental financial support, and fortifying digital infrastructure. In concluding remarks, the study highlights the potential hindrance posed by the uneven growth of cities to the success of pilot plans. To address this, the advocacy for government-led assistance programs is underscored, aiming at fostering comprehensive environmentally friendly development capable of reshaping traditional regional economies and catalyzing synchronized national growth. Business leaders are encouraged to embrace modernization and cultivate innovation to harness the benefits of smart cities, such as enhanced resource efficiency and reduced pollution. However, it is crucial to acknowledge certain limitations in the study, including constraints in panel data, challenges in accurately assessing city-level clean energy, and the intricate interplay of geographical and cultural factors. Future research endeavors are urged to pioneer innovative metrics for evaluating clean energy initiatives, incorporating considerations of urban geography and culture while delving into the cascading impacts of self-governing cities. This holistic approach holds the promise of addressing existing research gaps and significantly contributing to a nuanced understanding of smart city initiatives and their broader implications.

## CRediT authorship contribution statement

**Rui Ge:** Writing – review & editing, Writing – original draft, Visualization. **Shan Xu:** Software, Formal analysis. **Mirzat Ullah:** Data curation, Conceptualization. **Peter Mark:** Writing – original draft.

## Declaration of competing interest

The authors declare that they have no known competing financial interests or personal relationships that could have appeared to influence the work reported in this paper.
