# Supplementary material for: Assessing the impact of urban planning policies on renewable energy: A case a China using the DID estimation model
Source: Heliyon. 2024 Feb 24;10(5):e27099. doi: 10.1016/j.heliyon.2024.e27099 (PMC10923678; doi:10.1016/j.heliyon.2024.e27099)
Supplement: Multimedia component 1 [file mmc1.docx]

**Article Title:** Urban Planning Policy and Renewable Energy: A Chinese Perspective Research by Using DID model

Corresponding Author: Mr. Peter Mark

Dear Mr. Mark,

Your submission entitled "Urban Planning Policy and Renewable Energy: A Chinese Perspective Research by Using DID model" has been received by Heliyon. Please address the following:

| **Comments** | **Responses** |
| --- | --- |
| Please provide the cover letter for this submission | We have added the cover letter to the system |
| Please include list of author’s names, affiliations and their contact details in the cover letter of this submission | We have included all authors and their details in the cover letter |
| Please remove author identities [author name, affiliation, contact details] from manuscript file and provide these details in the cover letter of this submission | We have removed all author's details from the manuscript and added them in the cover letter provided to the system |
| Please include your full questionnaire as a supplementary file as part of your resubmission, this will be needed for the peer review process. (Fig 3 & 4 missing) | Figures numbering is edited and has been corrected as in sequence suggested by you. |
